# Understanding the Mechanisms of Cognitive Remediation on Recovery in People With Early Psychosis: A Mediation and Moderation Analysis

**DOI:** 10.1093/schbul/sbae021

**Published:** 2024-03-01

**Authors:** Rose Tinch-Taylor, Andrew Pickles, Dominic Stringer, Emese Csipke, Matteo Cella, Paul McCrone, Clare Reeder, Max Birchwood, David Fowler, Kathryn Greenwood, Sonia Johnson, Jesus Perez, Rosa Ritunnano, Andrew Thompson, Rachel Upthegrove, Jon Wilson, Alex Kenny, Iris Isok, Eileen M Joyce, Til Wykes

**Affiliations:** Institute of Psychiatry, Psychology and Neuroscience, King’s College London, London, UK; Institute of Psychiatry, Psychology and Neuroscience, King’s College London, London, UK; Institute of Psychiatry, Psychology and Neuroscience, King’s College London, London, UK; Institute of Psychiatry, Psychology and Neuroscience, King’s College London, London, UK; Institute of Psychiatry, Psychology and Neuroscience, King’s College London, London, UK; South London and Maudsley NHS Foundation Trust, London, UK; School of Health Sciences, University of Greenwich, London, UK; Institute of Psychiatry, Psychology and Neuroscience, King’s College London, London, UK; Warwick Medical School, University of Warwick, Coventry, UK; School of Psychology, University of Sussex, Brighton, UK; School of Psychology, University of Sussex, Brighton, UK; Faculty of Brain Sciences, University College London, London, UK; Cambridgeshire and Peterborough NHS Foundation Trust, Cambridge, UK; Warwick Medical School, University of Warwick, Coventry, UK; Warwick Medical School, University of Warwick, Coventry, UK; School of Psychology, University of Birmingham, Birmingham, UK; Norfolk and Suffolk NHS Foundation Trust, Norwich, UK; Patient Advisory Board, King’s College London, London, UK; Patient Advisory Board, King’s College London, London, UK; UCL Queen Square Institute of Neurology, University College London, London, UK; Institute of Psychiatry, Psychology and Neuroscience, King’s College London, London, UK; South London and Maudsley NHS Foundation Trust, London, UK

**Keywords:** mechanisms, cognitive, remediation, recovery, psychosis, mediation

## Abstract

**Background:**

To provide precision cognitive remediation therapy (CR) for schizophrenia, we need to understand whether the mechanism for improved functioning is via cognition improvements. This mechanism has not been rigorously tested for potential moderator effects.

**Study Design:**

We used data (*n* = 377) from a randomized controlled trial using CIRCuiTS, a therapist-supported CR, with participants from first-episode psychosis services. We applied structured equation modeling to test whether: (1) CR hours explain the goal attainment functional outcome (GAS) at posttreatment, (2) global cognitive improvement mediates GAS, and if (3) total symptoms moderate the CR hours to cognitive improvement pathway, and/or negative symptoms moderate the cognition to functioning pathway, testing moderator effects via the mediator or directly on CR hours to functioning path.

**Study Results:**

CR produced significant functioning benefit for each therapy hour (Coeff = 0.203, 95% CI 0.101–0.304, *P* < .001). The mediated path from CR hours to cognition and cognition to functioning was small and nonsignificant (Coeff = 0.014, 95% CI = −0.010, 0.037, *P* = .256). Total symptoms did not moderate the path to cognition (*P* = .211) or the direct path to outcome (*P* = .896). However, negative symptoms significantly moderated the effect of cognitive improvements on functioning (*P* = .015) with high negative symptoms reducing the functional gains of improved cognition.

**Conclusions:**

Although cognitive improvements were correlated with functioning benefit, they did not fully explain the positive effect of increased therapy hours on functioning, suggesting additional CR factors also contribute to therapy benefit. Negative symptoms interfere with the translation of cognitive improvements into functional gains so need consideration.

## Introduction

There is a well-established association between cognitive impairments and both current and future poor functional outcomes in people with a diagnosis of schizophrenia.^1^ Cognitive remediation (CR) was developed to reduce the impact of these cognitive difficulties with the assumption that this improvement will lead to functional benefits. CR has this assumption incorporated into its definition^2^ and clinical trials have included the effect of CR on both cognition and functioning as measures of efficacy. Treatment regulators have also adopted this assumption. Multiple meta-analyses have shown the durable benefits of CR on cognition, symptoms, and functioning,^3–7^ but few studies have tested mechanisms of their interrelationships. CR benefits could be enhanced by a better understanding of treatment mechanisms and especially whether improved cognition can account for functioning changes and whether any other variable changes the strength of this effect.

An ideal model of CR treatment effects examines both mediator and moderator variables. A mediator variable explains the relationship between a causal variable and an outcome variable, determining how or why something works, eg, improved cognition explains the relationship between CR treatment effects on functional outcome, whereas a moderator variable alters the strength or direction of the relationship between the 2 variables, eg, effect of symptoms. A systematic review of recent meta-analysis datasets^7,8^ supplemented by searches in PubMed and Web of Science, using (CR) AND (mediation OR moderation) AND (psychosis OR schizophrenia) AND (randomized controlled trial) as search terms, found 10 publications investigating mediation or moderation effects. Most referred^9^ to moderation only and many of those testing mediation looked only at correlations between cognitive improvement and functional outcome. Studies investigating treatment mechanisms using more stringent mediational models found partial mediation for cognition change on different functional outcomes,^9–16^ although the mediation effect was not large. Other studies had limitations including relying solely on correlations or multiple regression and most investigated single cognitive measures. More complex models with potential mediators *and* moderators are needed as they can expose treatment mechanisms more clearly.^17^ Methodological rigor is enhanced if moderation is combined with a mediation analysis since the decomposition into an effect of treatment on the mediator (action theory) and an effect of the mediator on outcome (conceptual theory) usually separates 2 theoretically different processes.^18^ These processes might be affected by different moderators.

The factors that may moderate how CR treatment contributes to improved functional outcomes^19^ include cognition at baseline, although the results are often contradictory with some showing that poorer cognition predicts more benefit and others showing the opposite. In addition, high levels of symptoms can also interfere with therapy engagement, especially if positive symptoms affect attention and concentration, and so may limit cognitive benefit, eg,^20,21^ although other evidence suggests that higher levels of symptoms are associated with more cognitive benefit.^7,22^ Even if cognition is improved, baseline negative symptoms, that encompass motivational difficulties and dysregulation in reward sensitivity, even if improved by CR,^23,24^ may still interfere with taking advantage of opportunities to make personal recovery progress.^7,25–31^ These potential symptom effects have not been consistently replicated.^32^

Two large recent meta-analyses^7,8^ demonstrated treatment benefits and good acceptability of CR in randomized controlled trials (RCTs). Therapy length had no effect, but there were important therapy characteristics that boosted CR benefits such as an active therapist, teaching strategies, and support for functional rehabilitation. Our recently completed Eclipse trial included these treatment elements enabling a detailed model of the effect of different mediators and moderators to be tested.^9^ Eclipse was conducted in United Kingdom NHS Early Intervention Services (EIS), which provide employment and education support alongside a comprehensive psychiatric service. CR was delivered using the software program CIRCuiTS, ie, therapist-supported and teaches strategies and transfer to real life. Using data from this trial we conducted a mediation and moderation analysis. We examined whether the effects of CR on cognitive gains were moderated by total symptoms, and whether negative symptoms interfere with the transfer of cognitive gains to functional benefits.

## Methods

### Design

Data for this secondary analysis comes from the ECLIPSE 4-arm multi-arm multistage (MAMS) RCT that examined 3 CR implementation methods that differed in the amount of therapist support received: independent therapy at home, group treatment, and one-to-one therapy, compared to treatment-as-usual (TAU) across 6 sites^33^ (see supplementary material for further information). The primary outcome was a personal recovery measure—the Goal Attainment Scale (GAS).^34^ CR was provided using the therapist-supported CIRCuiTS program^29^ that provides cognitive exercises as well as transfer tasks such as writing texts and going shopping (see^35^). The study found benefits for one-to-one and group treatment, but no benefit of independent therapy compared to TAU. Detail on the methods and results can be found in Wykes et al.^36^

**Fig. 1.**
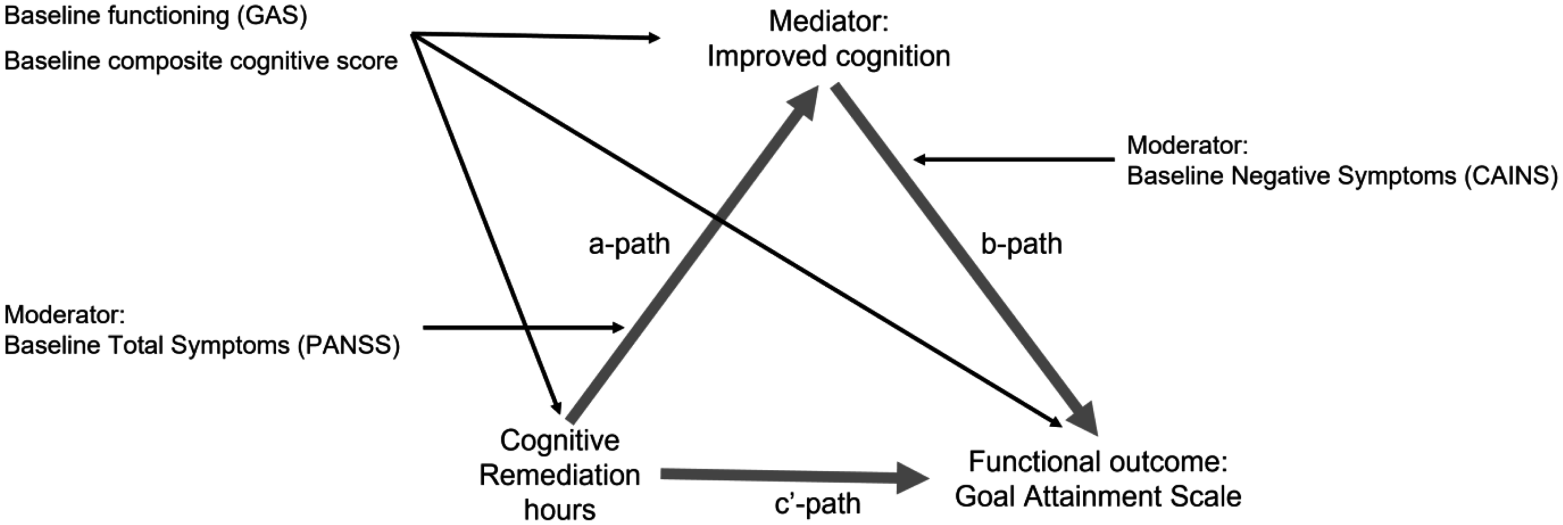
Mediation and moderation model.

An advantage of the Eclipse RCT data to test for mediation and moderation is that it compared different active conditions compared to previous mediation studies using single-group or parallel-arm designs assigning participants to CR or an active control.^11,13^ This Eclipse design offers the opportunity to explore subtle and important differences between different levels of therapist support as it included 4 arms that found that CR, provided by a therapist individually or in a group, benefitted functioning at 15 weeks posttreatment.^36^ The nature of these treatment arms allows the investigation of multiple-arm mediation and moderation effects (see figure 1). These were synthesized as part of a single moderated mediation model in stages. The first stage examined whether variation in CR benefit in any implementation arm could be explained solely by a single variable, hours of CR. The second examined whether CR-improved cognition explained change in functional outcome. The third investigated 2 potential moderators of the mediation process: total symptoms on cognitive benefits because they may boost or interfere with improvement; and negative symptoms because they interfere with taking advantage of environmental opportunities on the transfer of benefits from cognitive change to functioning outcome.

A mediator variable explains the relationship between a causal variable and an outcome variable, determining how or why something works. A moderator variable alters the strength or direction of the relationship between 2 variables. To test whether the mediation variable, improved cognition, explains the relationship between CR treatment effects on functional outcome, we estimated CR treatment effects on the potential mediator (change in cognition) and the effect of change in cognition on functioning with all other treatment effects summarized by a conditional direct CR effect path.

### Lived Experience Involvement

The therapy was co-developed with service users. For the ECLIPSE trial, different service users provided advice on the choice of outcome, overall design, and patient-facing documents and a further group of service users from our Patient Advisory Group provided critical review of analyses and some are authors of this article.

### Participants

Participants were aged between 16 and 45, at least 3 months from starting to use EIS, were clinically stable, and had a research diagnosis of non-affective psychosis.

The ECLIPSE trial was reviewed and approved by the Camden and Kings Cross RES NHS Committee (ref. number 15/LO/1960) and registered (ISRCTN14678860).

## Measures

### Therapy Dose

*CR hours* were measured using time in valid sessions where a valid session was defined as at least 10 min of completed therapy in each of the first 5 sessions and 20 min or more for the remaining sessions. The total number of hours was the total time in these valid sessions.

The *Mediator* is the change from baseline in the composite cognitive score (see supplementary material for further detail). The composite score was generated from baseline data and is calculated from 9 tests, 6 from the Cambridge Neuropsychological Test Automated Battery (CANTAB^37^) and 1 from each of the Rey Auditory Verbal Learning Test,^38^ Wisconsin Card Sorting Task,^39^ and Digit Span Backwards from the Wechsler Adult Intelligence Scale.^40^ To calculate the composite score: (1) some items were reverse scored so that higher was always better, (2) some scores were transformed to ensure all approximated a normal distribution, and (3) all individual cognitive scores were transformed into *Z* scores which were then trimmed to 3 or −3 before summing to get a composite score (see Wykes et al.^9^ for further detail). The cognitive measure was assessed at baseline and 15 weeks post-randomization.

*Moderators* were measured at baseline: (1) global psychopathology using the total score from the Positive and Negative Symptom Scale (PANSS^41^) and (2) negative symptoms using the total score from the Clinical Assessment Interview for Negative Symptoms (CAINS^42^).

The *primary functional outcome* was the GAS-weighted *T*-score at post-therapy (15 weeks post-randomization). GAS is a widely used self-report measure for psychosocial interventions,^43,44^ and is reliable and comparable to researcher reports.^34,45^ It was chosen as the most appropriate outcome based on advice from our Patient Advisory Board as it reflects the variability in the aspirations of users of EIS (see supplementary table S1, for examples of the goals). GAS scores are calculated by first identifying goals with the participant at baseline which are weighted on importance and difficulty identified in the scoring manual (see supplementary material) and then assesses whether these same goals are achieved after therapy.

## Statistical Methods

All analyses used Stata v17.0 sem (method mlmv) and gsem commands for structural equation modeling. Analyses of the GAS primary outcome covaried for baseline which is more powerful than the analysis of change scores.^46^ Similarly, we used the posttreatment cognitive outcome as the mediator and covaried for baseline cognition to analyze the effects of change in cognition. All models were estimated using maximum likelihood and assume that data were missing at random. This allowed for differential drop-out by treatment arm, baseline GAS, and randomization stratification factors. Where appropriate, randomization was exploited to set covariance terms between treatment assignment and baseline variables to zero. Model fit was assessed using chi-square and root-mean-square-error.

A simple and powerful analysis is possible if treatment differences across multiple trial arms can be reduced to a single common metric. CR hours were a plausible metric to represent the treatment dose as a recent study across different CR types and implementation methods demonstrated that increasing CR hours produce more cognitive gain.^47^ A preliminary analysis examined functional benefit across arms for each hour of CR, using Wald tests for an interaction between treatment arm and CR hours. In a second step, we tested whether the outcome differed between treatment arms after accounting for CR hours. Similar tests were undertaken to justify the exclusion of terms for interactions by site.

**Fig. 2.**
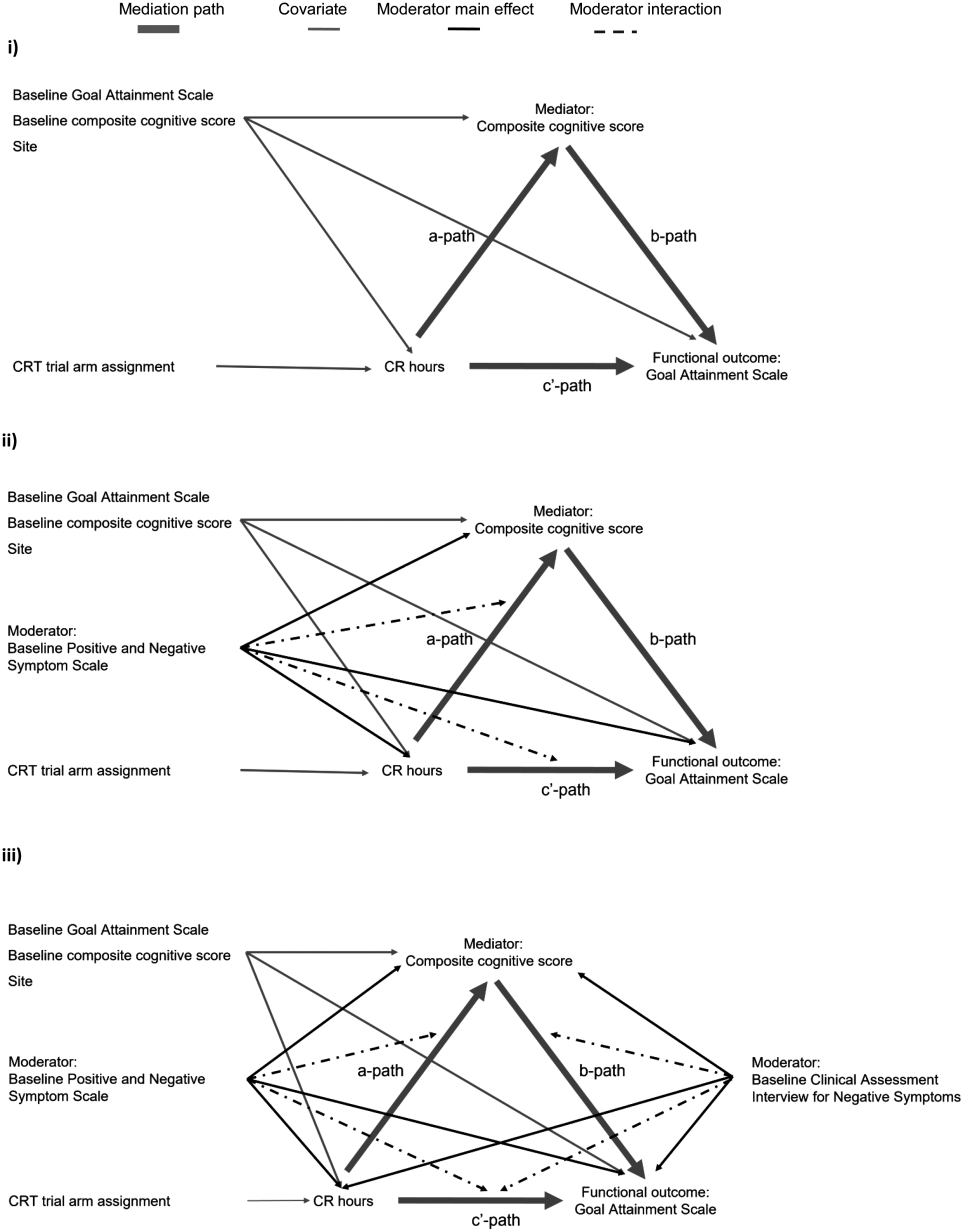
Assumed mediation/moderation of cognitive change (cognition score after CR, covarying for baseline score).

If the effects of treatment assignment occur through variations in CR hours, then a possible mediation model is shown in figure 2i. Following Landau, Emsley, and Dunn,^46^ we included baseline variables for both the mediator and outcome to minimize confounder bias. The mediator was measured using the cognitive outcome at posttreatment and covaried for baseline cognition to provide the cognitive gain produced by CR. As suggested by Shrout and Bolger,^48^ we used bootstrap (*n* = 2000 replicates) to estimate confidence intervals for the mediated effects (*a* times *b*).

Moderation by the continuous PANSS score on the *a*-path was estimated by including baseline PANSS score and the product of CR hours and baseline PANSS score in the prediction of change in endpoint cognition (figure 2ii), and on the *b*-path by the inclusion of baseline CAINS score and the product of cognition and baseline CAINS score in the prediction of change in endpoint GAS score (figure 2iii). Wald and likelihood ratio tests assessed whether *b*-path and *c*ʹ-path were significant after allowing for moderation of the *a*-path. To illustrate the moderator effects, models were re-estimated as a multi-group model with groups defined by a median split of the moderator variable, and model-predicted values extracted for plotting.

## Results

Table 1 presents background and treatment characteristics by trial arm and table 2 provides data on the primary outcome and control and moderator variables by trial arm that were used in the models. Supplementary table S2 shows outcome, control, and moderator variables data for all those entering the trial and supplementary table S3 shows further data on therapy hours.

**Table 1.** Baseline Characteristics and Therapy Hours for All Participants Entering the Study

	Group (*n* = 134)	Independent (*n* = 65)	One-to-one (*n* = 112)	TAU (*n* = 66)	All Participants (*n* = 377)
Age at consent Mean (SD)	25.19 (5.91)	25.92 (5.56)	26.39 (6.72)	25.14 (5.55)	25.67 (6.05)
Sex *N* (%)					
Men	90 (67.2%)	47 (72.3%)	83 (74.1%)	55 (83.3%)	275 (72.9%)
Women	44 (32.8%)	18 (27.7%)	29 (25.9%)	11 (16.7%)	102 (27.1%)
Ethnicity *N* (%)					
White	59 (44.0%)	32 (49.2%)	57 (50.9%)	37 (56.1%)	185 (49.1%)
Black (African, Caribbean)	41 (30.6%)	20 (30.8%)	27 (24.1%)	17 (25.8%)	105 (27.9%)
Asian (Bangladeshi, Indian, Pakistani)	14 (10.4%)	4 (6.2%)	18 (16.1%)	7 (10.6%)	43 (11.4%)
Other	20 (14.9%)	9 (13.8%)	9 (8.0%)	5 (7.6%)	43 (11.4%)
Employment status *N* (%)					
Unemployed	87 (64.9%)	44 (67.7%)	77 (68.8%)	42 (63.6%)	250 (66.3%)
Primary childcare giver	0 (0.0%)	3 (4.6%)	0 (0.0%)	1 (1.5%)	4 (1.1%)
In full-time education	27 (20.1%)	8 (12.3%)	10 (8.9%)	7 (10.6%)	52 (13.8%)
Part-time employed	9 (6.7%)	5 (7.7%)	13 (11.6%)	9 (13.6%)	36 (9.5%)
Full-time employed	11 (8.2%)	5 (7.7%)	12 (10.7%)	7 (10.6%)	35 (9.3%)
Living situation *N* (%)					
Own property (private, rented)	40 (29.9%)	23 (35.4%)	32 (28.6%)	21 (31.8%)	116 (30.8%)
Parental home	79 (59.0%)	30 (46.2%)	60 (53.6%)	36 (54.5%)	205 (54.4%)
Temporary accommodation	4 (3.0%)	4 (6.2%)	4 (3.6%)	3 (4.5%)	15 (3.9%)
Supervised Group Home	6 (4.5%)	4 (6.2%)	8 (7.1%)	3 (4.5%)	21 (5.6%)
Supervised Hostel	5 (3.7%)	4 (6.2%)	7 (6.2%)	3 (4.5%)	19 (5.0%)
* Missing*	0 (0.0%)	0 (0.0%)	1 (0.9%)	0 (0.0%)	1 (0.3%)
Relationship status *N* (%)					
Single	120 (89.6%)	54 (83.1%)	98 (87.5%)	59 (89.4%)	331 (87.8%)
Living with partner	7 (5.2%)	6 (9.2%)	9 (8.0%)	6 (9.1%)	28 (7.4%)
Married/Same-sex civil partnership	4 (3.0%)	4 (6.2%)	4 (3.6%)	0 (0.0%)	12 (3.2%)
Separated/Divorced	3 (2.2%)	0 (0.0%)	1 (0.9%)	1 (1.5%)	5 (1.3%)
* Missing*	0 (0.0%)	1 (1.5%)	0 (0.0%)	0 (0.0%)	1 (0.3%)
CR Hours Mean (SD)	14.45 (12.72)	8.84 (9.90)	19.38 (12.82)	0 (0.00)	14.54 (12.87)

**Table 2.** Complete Cases Data on Variables Used in the Models

Variable	Group	Independent	One-to-One	TAU	Total
Cognitive composite score for complete data cases					
* N*	46	20	45	24	135
* *Baseline Mean (SD)	−0.57 (5.80)	−0.78 (5.36)	0.77 (5.14)	0.57 (5.23)	0.05 (5.41)
* *Post-therapy Mean (SD)	−0.51 (5.84)	−0.00 (4.86)	1.04 (5.25)	−0.65 (4.57)	0.06 (5.29)
GAS total score for complete data cases					
* N*	91	36	82	43	252
* *Baseline Mean (SD)	33.57 (4.52)	34.15 (4.54)	32.29 (5.03)	33.98 (4.07)	33.31 (4.66)
* *Post-therapy Mean (SD)	52.17 (11.12)	46.74 (9.64)	50.9 (12.08)	46.60 (12.20)	50.02 (11.61)
Baseline PANSS composite score					
* N*	132	64	111	64	371
* *Mean (SD)	55.24 (14.19)	59.66 (19.18)	57.35 (16.45)	55.64 (14.14)	56.70 (15.84)
Baseline CAINS composite score					
* N*	134	65	109	65	373
* *Mean (SD)	17.42 (9.31)	18.65 (9.68)	18.62 (9.56)	17.25 (8.44)	17.95 (9.29)

### Does Functional Benefit Occur Through CR Treatment Hours?

**Fig. 3.**
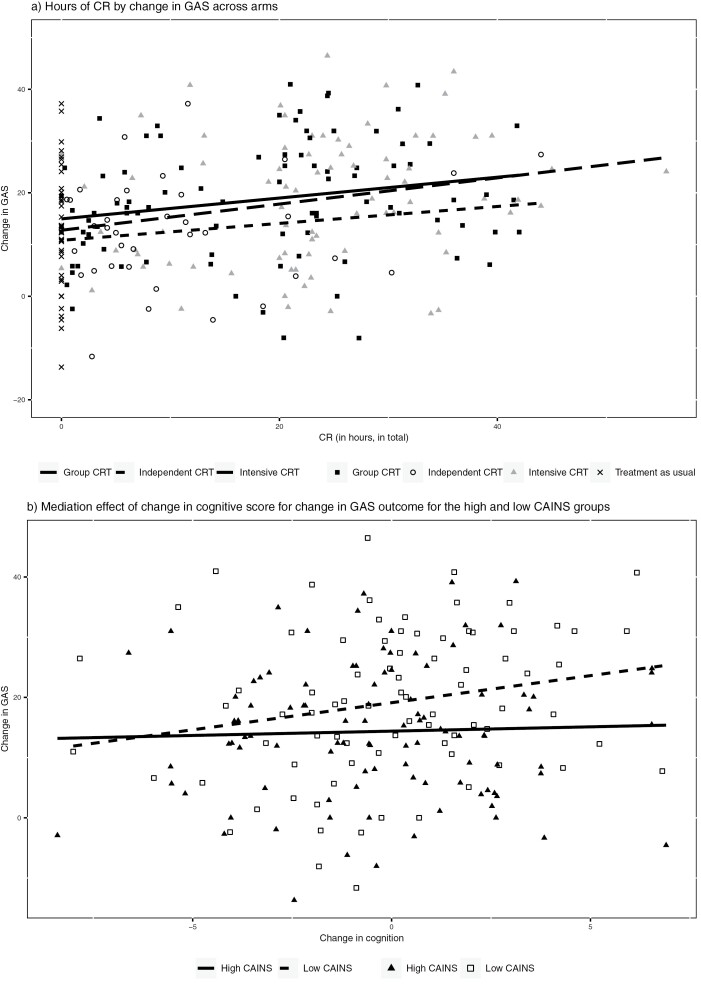
Change in GAS across arms by: (a) CR hours and (b) mediation effect of change in cognitive score.

Although there was variation in CR hours between implementation methods there was a similar relationship between CR hours and change in GAS for each active treatment arm (see figure 3a). Wald tests of interaction terms between implementation method and CR hours on GAS outcome showed no overall difference (*c*^2^(3) = 4.47, *P* = .215), and there was no significant difference between methods with a more therapist input (individual and group) and those with less (independent and TAU) (*c*^2^(1) = 0.10, *P* = .753). Wald tests also justified the exclusion of interaction terms between site and treatment arm on CR hours (*c*^2^(12) = 12.77, *P* = .386), and between site and treatment arm on GAS outcome (*c*^2^(12) = 13.53, *P* = .332). The effect on the GAS-T score can therefore be simplified to a difference in CR hours, not just for explaining the differences among the CR arms, but also for comparing CR methods to the no-CR group.

This simplification allowed us to fit the mediation model in figure 2i. Wald tests confirmed that implementation methods did not predict the cognitive mediator (*c*^2^(3) = 2.88, *P* = .411), nor did they predict the GAS functional outcome (*c*^2^(3) = 4.94, *P* = .176) once the CR hours variable was included. The mediation model fitted well (Root Mean Square Error of Approximation (RMSEA) = 0.00; *c*^2^(27) = 23.314, *P* = .668). The estimate of the standardized conditional direct effect of CR hours on GAS outcome was highly significant (*P* < .001; 0.203 CI 0.101–0.304), however, the path from CR hours to cognition was not significant (0.024, CI −0.007 to 0.054, *P* = .125). The effect along the *b*-path of change in cognition on change in the functional outcome was significant (0.572, CI 0.049–1.095, *P* = .032). See supplementary table S4 for the mediation model detail. This gave a mediated path (*a* times *b*), that was small and nonsignificant (0.014, 95% CI = −0.010 to 0.037, *P* = .256).

A test for moderated mediation of the *a*-path by the baseline PANSS score was nonsignificant (*c*^2^(1) = 1.56, *P* = .0.211; see figure 2ii), and similarly for the *c*ʹ path (*c*^2^(1) = 0.02, *P* = .896). The baseline CAINS score had no effect on either CR hours or the cognitive composite, but significantly moderated the effect of the cognitive composite on the GAS outcome (see table 3). Figure 3b shows that for participants with high levels of negative symptoms (CAINS median >16) there was no significant association between improved cognition and improved functional outcome. For those with fewer negative symptoms (median CAINS score ≤16), improved cognition was associated with gains in the functional outcome.

**Table 3.** Moderated Mediation

	Unstandardized Estimate (95% CI)	*P*-value
CAINS Main Effect Prediction of		
CR hours	0.067 (−0.084,0.218)	0.386
Composite cognitive score	−0.026 (−0.077, 0.026)	0.327
GAS-T score	−0.064 (−0.365, 0.237)	0.677
CAINS Moderation		
*b*-path	−0.039 (−0.069, −0.008)	0.014
*c*ʹ-path	−0.003 (−0.017, 0.010)	0.642

## Discussion

### Does treatment time affect CR benefits?

The ECLIPSE RCT reported the benefits of CR delivered by a therapist to individuals or groups of patients on the GAS-T functional outcome.^36^ The design of this RCT meant that CR sessions were offered over a fixed period of 12 weeks even though some may have had engagement difficulties. For instance, if the person did not attend for a week these missed sessions were not reinstated. There was also some difference between arms in the engagement rates. This produced variability in the CR dose delivered and allowed us to investigate the dose-response relationship between time on the CR tasks and change in GAS-T. We demonstrated how the effect of CR hours was similar regardless of whether they were delivered individually, in groups or independently, and there were no significant differences between trial arms on GAS once it had been accounted for. This is an important result as it indicates that we need to concentrate on engagement to achieve benefits and so our approach to therapy completion time in clinical services needs to be flexible. This is similar to the results from a recent study^47^ that found that if more sessions were offered there was a larger improvement in some cognitive functions, specifically executive function and processing speed. We do not know if specific cognitive tests improved more than others with hours of therapy in our study as we used a composite score, but this seems likely. The CR hours measure was much greater when a therapist was involved, but if individuals engaged with independent therapy and completed enough sessions then we would expect a measurable improvement.

### Does cognitive change affect functional improvement?

Our mediation analysis suggested that while cognitive changes during treatment were associated with changes in the functional outcome, there was no dose-response relationship between CR hours and cognitive change. There was also no significant mediation effect of cognition between CR hours and functioning. CR effects must therefore be through other paths, summarized by the significant conditional direct effect.

There are several potential explanations of this lack of effect through cognition in our study. The first is that the significant effects of cognition on functioning that have been found in other studies^10,13^ test several cognitive measures, whereas we used a composite cognitive score that gathered all potential improvements in cognitive tests, but it may have hidden some significant cognitive domain relationships.^7,49^ Some studies also used proxy functional capacity measure known to be closely related to cognition rather than recovery outcomes and therefore suggest a direct relationship when one was absent. We carried out our study during the Covid-19 period when lockdown meant there were fewer opportunities for practicing some skills necessary to complete a goal, eg, for shopping and social activity goals. Clearly, providing formal psychosocial rehabilitation might have offered these opportunities although as there were varied goals providing this tailored rehabilitation would have been onerous. Another potential explanation is that we have improved self-efficacy and reduced defeatist beliefs.^50,51^ As we did not measure either we cannot be certain, but the method of CR implemented does allow for much positive and constructive feedback that may have altered these factors. Alternatively, and our preferred explanation, is that change in another aspect of cognition—metacognition—may underlie the improvements in functional outcomes. Teaching metacognition is known to improve skills in educational contexts^52^ and for this reason we adopted it into our model of CR therapy and embedded this into CIRCuiTS.^35^ Learning metacognitive skills is built into the CR program including when and how to implement strategies to solve real-world tasks through self-reflection before, within and at the end of each task. Metacognition is related to functioning outcomes in schizophrenia.^53–55^ We know the program does improve metacognitive skills^56^ and that metacognition predicts functional outcome^57,58^ and when included removes the effects of cognition on outcome. We therefore believe that the most parsimonious explanation is that metacognitive improvement affects outcome.

We examined moderated mediation by investigating whether high or low PANSS scores (overall psychopathology) influenced how CR hours could change cognition (*a*-path moderation) and whether the CAINS scores (negative symptoms) influenced how the benefits of cognitive change were translated into functional benefits (*b*-path moderation). While no effect was found for the PANSS, similar to others,^59^ those with high CAINS scores did not benefit from changes in cognition, in contrast to those with lower scores. This suggests that negative symptoms can interfere with mobilizing improvements in general cognition to achieve a desired functional outcome. This is a potentially important variable to consider for personalizing CR as those with higher negative symptoms may need longer CR or further support to use community opportunities to learn or re-learn social and other skills. As well as this clinical implication for CR implementation, there is also a further regulation issue. Functional change is a key indicator of the benefit of CR therapy, and we may miss therapy efficacy by only testing a simple model. This analysis has shown that other variables need to be considered, potentially change in metacognition and negative symptoms to understand treatment outcomes.

## Strengths and Limitations

We report a mediation analysis for one of the largest CR trials, and the randomized treatment allocation enhanced our ability to attribute causal effects to the estimated associations. We made the standard assumptions of most mediation analyses of linearity, normality, homogeneity of error variance, independence of errors, and no measurement error in CR hours and the cognitive mediator. More frequent, repeated assessment of mediator and outcome allows us to account for measurement error and more convincing causal attribution of effects.^60^ However, the quite different measurement bases of the intervention (from observation), mediator (from experimental testing), and outcome (from participant report for an outcome based on each participants own priorities) likely reduced the risk of estimated effects being contaminated by correlated measurement error.

To avoid power loss associated with multiple-testing and to increase internal and external validity^61^ we chose to investigate a single mediator and 2 a priori specified potential moderators. Exploration of other mediated paths, such as through metacognition, and other potential CR moderators, such as skills achieved in therapy, therapeutic alliance, or specific cognitive difficulties that may interfere with therapy benefits, may also be worthwhile.

## Conclusion

As in other studies we found a relationship between change in cognition and functioning, but we did not find a significant mediated effect. This might be because of the nature of the cognitive composite measure, the type of functioning outcome or the model of CR embedded in our software. We did, however, discover that high levels of negative symptoms at the beginning of therapy interfered with the translation of cognitive gains to functional benefit. This has implications for clinical services and suggests that individual with high levels of negative symptoms may benefit from an increased number of CR therapy hours and perhaps increased opportunities to support the transfer of therapy gains into valued daily activities and recovery goals. We also suggest that trials of CR therapies, including pharmaceutical therapies, should also consider the potential moderators of success so we do not miss potentially advantageous treatments.

## Supplementary Material

Supplementary material is available at https://academic.oup.com/schizophreniabulletin/.

sbae021_suppl_Supplementary_Material

## Open Access

For the purposes of open access, the author has applied a Creative Commons Attribution (CC BY) licence to any Accepted Author Manuscript version arising from this submission.
