# Supplementary material for: Understanding the Mechanisms of Cognitive Remediation on Recovery in People With Early Psychosis: A Mediation and Moderation Analysis
Source: Schizophr Bull. 2024 Mar 1;50(6):1371–81. doi: 10.1093/schbul/sbae021 (PMC11548933; doi:10.1093/schbul/sbae021)
Supplement: sbae021_suppl_Supplementary_Material [file sbae021_suppl_supplementary_material.docx]

**Supplementary information**

Contents

[Measures 1](#_Toc123738230)

[1. Goal Attainment Score (GAS) 1](#_Toc123738231)

[2. Composite cognitive score 2](#_Toc123738232)

[Results 4](#_Toc123738233)

[Table 1s - Structural Equation Model results for mediation model without moderators. 4](#_Toc123738234)

# Measures

## **Goal Attainment Score (GAS)**

**What is GAS?**

GAS is a method of scoring the extent to which patient’s individual goals are achieved in the course of intervention. In effect, each patient has their own outcome measure but this is scored in a standardised way as to allow statistical analysis.

Traditional standardised measures include a standard set of tasks (items) each rated on a standard level. In GAS, tasks are individually identified to suit the patient, and the levels are individually set around their current and expected levels of performance.

**How is GAS rated?**

An important feature of GAS is the ‘a priori ‘ establishment of criteria for a ‘successful’ outcome in that individual, which is agreed with the patient and family before intervention starts so that everyone has a realistic expectation of what is likely to be achieved, and agrees that this would be worth striving for. Each goal is rated on a 5-point scale, with the degree of attainment captured for each goal area:

If the patient achieves the expected level, this is scored at 0.

If they achieve a better than expected outcome this is scored at:

+1 (somewhat better)

+2 (much better)

If they achieve a worse than expected outcome this is scored at:

-1 (somewhat worse) or

-2 (much worse)

**How is GAS calculated?**

The **GAS weighted T-score** was calculated by applying the following formula (as specified in the GAS Practical Guide [19]):

$$\frac{10\sum{(w}_{i}x_{i})}{{[\left( 1-\rho\right)\sum{w_{i}}^{2}+ \rho\left( {\sum{(w}_{i})}^{2} \right)]}^{\frac{1}{2}}}$$

Where:

$w_{i}$ = the weight assigned to the ith goal

$x_{i}$ = the numerical value achieved (between -2 and +2)

$\rho$ = the expected correlation of the goal scales, we will use 0.3 as recommended by Kirusek and Sherman [10] as this is most common approximation.

## **Composite cognitive score**

***Cambridge Neuropsychological Test Automated Battery*** (CANTAB, Barnett et al., 2010) is a neuropsychological test presented on a touchscreen, mirroring the seven domains evaluated by MATRICS (attention/vigilance, working memory spatial, visual learning, speed of processing, verbal learning, social cognition, and reasoning/problem solving). Studies showed good levels of test-retest reliability: r>0.8 for some measures of visual learning and r>0.7 for spatial working memory and executive functioning (Lowe and Rabbitt 1998).

The **composite cognitive outcome** is made up from the following cognitive measures from both the CANTAB and supplemented by individual measures that have been sensitive to cognitive remediation from the baseline scores of this sample. Items (in italics below) were reverse scored (lower is better) and some items were transformed to be approximately normally distributed (see below). All items were then Z-transformed into Z scores (to give items equal weight). These Z-scores were then trimmed (to 3 or -3 if they exceeded these values) before summing to get a composite score. The composite score was pro-rated (the missing item replaced by mean of other Z-transformed trimmed items) if 1 of the below items was missing and missing otherwise (if 2 or more items missing).

**CANTAB measures**

- Attention switching - Total correct responses for congruent and incongruent trials.
- Rapid visual information processing continuous performance test - Signal detection measure of a subject's sensitivity to the target sequence (string of three numbers), regardless of response tendency. This was first reverse scored and log-transformed.
- *Simple and 5 choice reaction time - Median 5 choice reaction time correct. This was reverse scored.*
- One touch Stockings of Cambridge Test of Planning - Number of problems solved on first choice.
- *Spatial Working Memory - Between Search Errors: Number of errors across whole task - revisiting boxes which have already been found to contain a token. This was reverse scored.*
- *Paired Associate Learning - Adjusted Errors Performance across whole task estimated for all stages if any were terminated early. This was reverse scored.*

**Individual neuropsychological tests**

- Rey Auditory Verbal Learning Test (RAVLT; (Rey and Osterreith 1993) – Sum of all attempts
- *Wisconsin Card Sorting Task* (*WCST; (Heaton RK 1993) – Perseverative errors. This was first reverse scored and log-transformed.*
- Wechsler Adult Intelligence Scale (WAIS; (Wechsler 1997) Digit span Backward raw score.

**Intervention modes**

More information on CIRCuiTS^TM^ can be found on https://www.circuitstherapyinfo.com/ It was developed by Til Wykes and Clare Reeder based on pedagogical theory that it is important to teach new skills by supporting metacognition as this allows the transfer of cognitive skills to new problems and situations (see Wykes and Reeder, 2005). Previous studies have shown that metacognition is improved with CIRCuiTS^TM^ (see (Cella et al. 2019; Hyde et al. 2020; Hiekkala-Tiusanen et al. 2019; Van Duin et al. 2021; Thomas and Rusten 2019; Palumbo et al. 2019; Drake et al. 2014)). CIRCuiTS^TM^ has been tested previously both in studies by the developers and others and have shown improvements in cognition and functioning (e.g(Hyde et al. 2020; Hiekkala-Tiusanen et al. 2019; Van Duin et al. 2021; Thomas and Rusten 2019; Palumbo et al. 2019; Drake et al. 2014).

The chosen intervention modes are those traditionally adopted in different cognitive remediation studies but not compared in a single study.

CIRCuiTS^TM^ engages cognition, metacognition, and the transfer of these skills to functioning both within the therapy and with the help of a therapist. The therapy includes cognitive tasks (as in other software programmes) but also includes exercises where the cognitive skills (planning, strategy use and metacognitive awareness and regulation) are used to engage with real life activities such as shopping, cooking, and travelling on public transport to aid transfer. It also allows therapeutic conversations about how strategies and planning (metacognition) will help complete the exercises, but as the software has built-in transfer opportunities to daily life activities and toward personal goals it may not need a therapist.

(a) One-to-one CR. Participants receive 10.5 weeks of twice weekly therapy, up to 42 h in total, with sessions lasting between 60 and 180 min, split into three parts: (1) 20–60 min of CR with a therapist; (2) 20–60 min of in-vivo transfer work (i.e., putting CR strategies into real life) with a therapist; (3) 20–60 min of independent CR, set up by the therapist on-site, or off-site in the service user’s own time. A session is ‘valid’ if it lasts a minimum of 20 min.

(b) Group CR. Participants are offered 14 weeks of 3 times weekly group therapy (up to 42 h of CR). Sessions last up to 90 min, with attendance for at least 20 min considered as a completed session. Groups have four participants and one therapist. The sessions begin and end with group activities related to goal setting and metacognition. For the remainder of the session, service users work independently, with the therapist offering help and support on an as-needed basis.

(c) Independent CR. Participants receive one individual session with the therapist for orientation followed by up to 41 sessions when they work independently (up to 42 h of CR in total). To support the independent sessions, the therapist will offer telephone contact or attendance at drop-in sessions on an as-needed basis to address any questions or problems (but not exceeding 1 h contact time per fortnight). A session is ‘valid’ if it lasts a minimum of 20 min.

(d) Treatment as usual. This is the set of standard interventions offered by the treating team without restrictions. It involves clinical contact on a daily, weekly, or monthly basis depending on recovery as well as educational or employment programmes, other psychological therapies, e.g., cognitive behaviour therapy for psychosis and medical treatments, including drug therapies.

**Table 1s Examples of SMART Goals in the GAS scaling**

| **SMART goal** | **Importance** | **Difficulty** |
| --- | --- | --- |
| Plan, prepare and practice keyboard for 30 minutes every other day | 2. Moderately | 1. A little |
| Read psychology books online for 30 minutes per week | 1. A little | 3. Very |
| Plan, prepare or read for homework/classes for 1.5 hours everyday | 3. Very | 2. Moderately |
| Learn to drive by planning and arranging lessons and practice theory test | 3. Very | 2. Moderately |
| Spend 30 minutes 3x per week to plan clothes and to do hair in the morning | 2. Moderately | 1. A little |
| Eat a healthy breakfast 2-3 times per week and get up early | 3. Very | 2. Moderately |
| Contact friends 2x per week for 30 minutes | 2. Moderately | 1. A little |
| Look for plumbing courses for 1 hour per week and update CV | 2. Moderately | 2. Moderately |
| Look for jobs online three times a week for one hour each time. | 1. A little | 2. Moderately |
| Write down distractions and go through a mental ''walking checklist'' | 2. Moderately | 2. Moderately |
| Going to the shops alone | 3. Very | 3. Very |
| Save £100 per month | 3. Very | 2. Moderately |
| Look for work 3x per week for 1 hour | 2. Moderately | 3. Very |
| Plan time to go out 2x per week to look for work and hand out CV | 1. A little | 2. Moderately |
| Eat 3 meals per day and have 5 home cooked meals per week | 3. Very | 2. Moderately |
| 1x a week look online to find events | 2. Moderately | 1. A little |
| Twice a week go to the gym for 2 hours and twice a week read about fitness and nutrition for 2 hours | 3. Very | 2. Moderately |
| Play the guitar 1x a week for 1 hour | 1. A little | 2. Moderately |
| To organise & cook at least 1 healthy meal a day | 3. Very | 2. Moderately |
| To exercise at least 3 times a week | 2. Moderately | 2. Moderately |

**Table 2s Baseline and post treatment scores for all participants entering the trial**

| **PANSS Total score** Mean (SD) | 55.24 (14.19) | 59.66 (19.18) | 57.35 (16.45) | 55.64 (14.14) | 56.70 (15.84) |
| --- | --- | --- | --- | --- | --- |
| **CAINS Total score** Mean (SD) | 17.42 (9.31) | 18.65 (9.68) | 18.62 (9.56) | 17.25 (8.44) | 17.95 (9.29) |
| **Composite cognitive score*** Mean (SD) | 0.11 (4.92) | -0.34 (5.77) | 0.94 (5.09) | -0.40 (6.01) | 0.18 (5.33) |
| **GAS T-score** Mean (SD) | 33.46 (4.58) | 33.93 (4.37) | 32.48 (5.11) | 33.78 (4.39) | 33.31 (4.69) |
| **Post Baseline Measures at Trial Endpoint** | | | | | |
| **CR Hours**Mean (SD) | 14.45 (12.72) | 8.84 (9.90) | 19.38 (12.82) | 0 (0.00) | 14.54 (12.87) |
| **Cognitive Composite score***Mean (SD) | -0.18 (5.50) | 0.84 (5.60) | 0.61 (5.19) | -0.53 (4.73) | 0.17 (5.26) |
| **GAS T-score**Mean (SD) | 52.14 (11.06) | 46.74 (9.64) | 50.87 (12.01) | 46.60 (12.20) | 50.02 (11.56) |

**Table 3s Hours of therapy**

| Table 3s Therapy adherence | | | | |
| --- | --- | --- | --- | --- |
|  | **Independent** | **Group** | **One-to-One** | **Total** |
| **N (%)** | 65 (20·9%) | 134 (43·1%) | 112 (36·0%) | 311 (100·0%) |
| **Valid sessions of therapy attended** Mean (SD) | 11·59 (12·33) | 12·49 (10·87) | 15·59 (10·41) | 13·4 (11·1%) |
| **Number of hours of therapy attended** Mean (SD) | 8·84 (9·90) | 14·45 (12·72) | 19·38 (12·82) | 15·1 (12·8%) |
| **Received at least one therapy session** Yes N (%) | 63 (96·9%) | 122 (91·0%) | 103 (92·0%) | 288 (92·6%) |
| **Received minimum dose of 20 therapy sessions** Yes N (%) | 15 (23·1%) | 40 (29·9%) | 41 (36·6%) | 96 (30·9%) |
| **Drop-out - 5 sessions or less (%)** | 29 (45·3%) | 50 (37·6%) | 24 (21·6%) | 108 (33·4%) |
| **Received 20 session minimum dose after drop-out** | 15 (42·9%) | 40 (48·2%) | 41 (47·1%) | 96 (46·8%) |

# Results

## **Table 4s - Structural Equation Model results for mediation model without moderators.**

| **Table 4s: Mediation model (Figure 2i)** | | |
| --- | --- | --- |
|  | **Unstandardized Estimate (95% CI)** | **p-value** |
| **CR hours** |  |  |
| Independent CR | 8.795 (5.055,12.534) | 0.000 |
| Intensive CR | 19.179 (15.826,22.532) | 0.000 |
| Group CR | 14.338 (11.109,17.567) | 0.000 |
| Site 2 | -3.291 (-7.434,0.852) | 0.119 |
| Site 3 | -3.212 (-7.866,1.442) | 0.176 |
| Site 4 | 2.204 (-2.527,6.936) | 0.361 |
| Site 5 | 1.305 (-2.409,5.019) | 0.491 |
| Site 6 | -0.373 (-4.253,3.507) | 0.851 |
| Baseline Composite cognitive score | 0.012 (-0.208,0.232) | 0.916 |
| Baseline GAS-T score | -0.012 (-0.268,0.244) | 0.927 |
| **Composite cognitive score** |  |  |
| CR hours | 0.024 (-0.007,0.054) | 0.125 |
| Site 2 | 1.715 (0.238,3.192) | 0.023 |
| Site 3 | 2.801 (0.984,4.619) | 0.003 |
| Site 4 | 0.866 (-0.817,2.549) | 0.313 |
| Site 5 | 1.36 (-0.023,2.742) | 0.054 |
| Site 6 | 0.89 (-0.53,2.311) | 0.219 |
| Baseline Composite cognitive score | 0.84 (0.761,0.918) | 0.000 |
| Baseline GAS-T score | 0.013 (-0.077,0.103) | 0.777 |
| **GAS-T score** |  |  |
| CR hours | 0.203 (0.101,0.304) | 0.000 |
| Composite cognitive score | 0.572 (0.049,1.095) | 0.032 |
| Site 2 | -3.579 (-8.457,1.299) | 0.150 |
| Site 3 | 4.349 (-1.562,10.259) | 0.149 |
| Site 4 | 0.728 (-4.904,6.36) | 0.800 |
| Site 5 | 1.286 (-3.164,5.735) | 0.571 |
| Site 6 | -0.836 (-5.417,3.745) | 0.721 |
| Baseline Composite cognitive score | -0.239 (-0.748,0.27) | 0.358 |
| Baseline GAS-T score | 0.45 (0.152,0.747) | 0.003 |
